# Supplementary material for: Transcriptional Alterations in X-Linked Dystonia–Parkinsonism Caused by the SVA Retrotransposon
Source: Int J Mol Sci. 2022 Feb 17;23(4):2231. doi: 10.3390/ijms23042231 (PMC8875906; doi:10.3390/ijms23042231)
Supplement: Supplementary file 1 [file ijms-23-02231-s001.zip › ijms-1534084-supplementary.pdf]

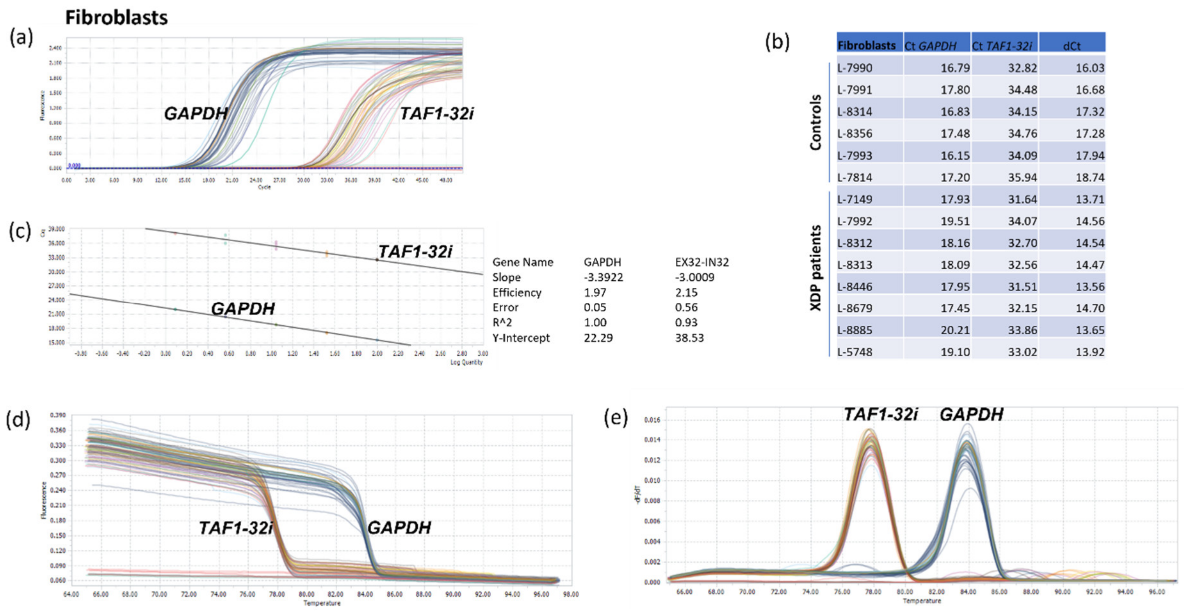

	Fibroblasts	Ct *GAPDH*	Ct *TAF1-32i*	dCt
Controls	L-7990	16.79	32.82	16.03
	L-7991	17.80	34.48	16.68
	L-8314	16.83	34.15	17.32
	L-8356	17.48	34.76	17.28
	L-7993	16.15	34.09	17.94
	L-7814	17.20	35.94	18.74
XDP patients	L-7149	17.93	31.64	13.71
	L-7992	19.51	34.07	14.56
	L-8312	18.16	32.70	14.54
	L-8313	18.09	32.56	14.47
	L-8446	17.95	31.51	13.56
	L-8679	17.45	32.15	14.70
	L-8885	20.21	33.86	13.65
	L-5748	19.10	33.02	13.92

Figure S1. Quality control information for *GAPDH* and *TAF1-32i* qPCRs performed in fibroblasts. (**a**) amplification plots; (**b**) table with the individual Ct values for both genes, as well as dCt values (Ct *TAF1-32i* – Ct *GAPDH*); (**c**) standard curves with information including their efficiencies; (**d**) melting curves representing raw data, i.e., plotting fluorescence over temperature; (**e**) melting curves representing negative first derivatives (-dF/dT).

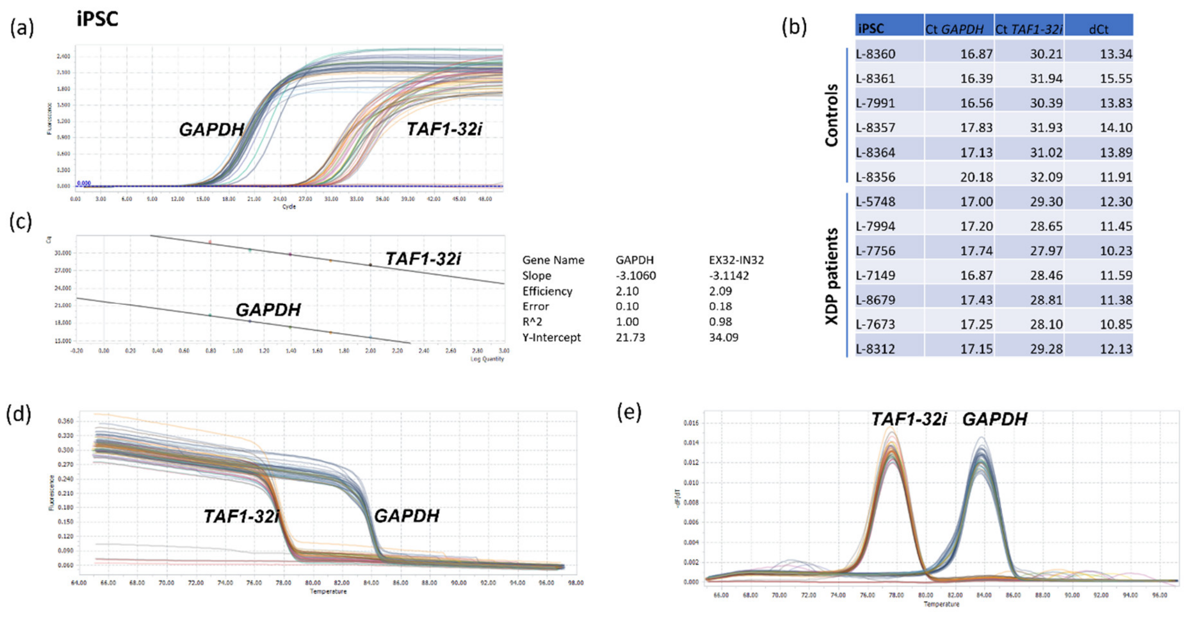

	iPSC	Ct *GAPDH*	Ct *TAF1-32i*	dCt
Controls	L-8360	16.87	30.21	13.34
	L-8361	16.39	31.94	15.55
	L-7991	16.56	30.39	13.83
	L-8357	17.83	31.93	14.10
	L-8364	17.13	31.02	13.89
	L-8356	20.18	32.09	11.91
XDP patients	L-5748	17.00	29.30	12.30
	L-7994	17.20	28.65	11.45
	L-7756	17.74	27.97	10.23
	L-7149	16.87	28.46	11.59
	L-8679	17.43	28.81	11.38
	L-7673	17.25	28.10	10.85
	L-8312	17.15	29.28	12.13

Figure S2. Quality control information for *GAPDH* and *TAF1-32i* qPCRs performed in iPSCs. (**a**) amplification plots; (**b**) table with the individual Ct values for both genes, as well as dCt values (Ct *TAF1-32i* – Ct *GAPDH*); (**c**) standard curves with information including their efficiencies; (**d**) melting curves representing raw data, i.e., plotting fluorescence over temperature; (**e**) melting curves representing negative first derivatives (-dF/dT).

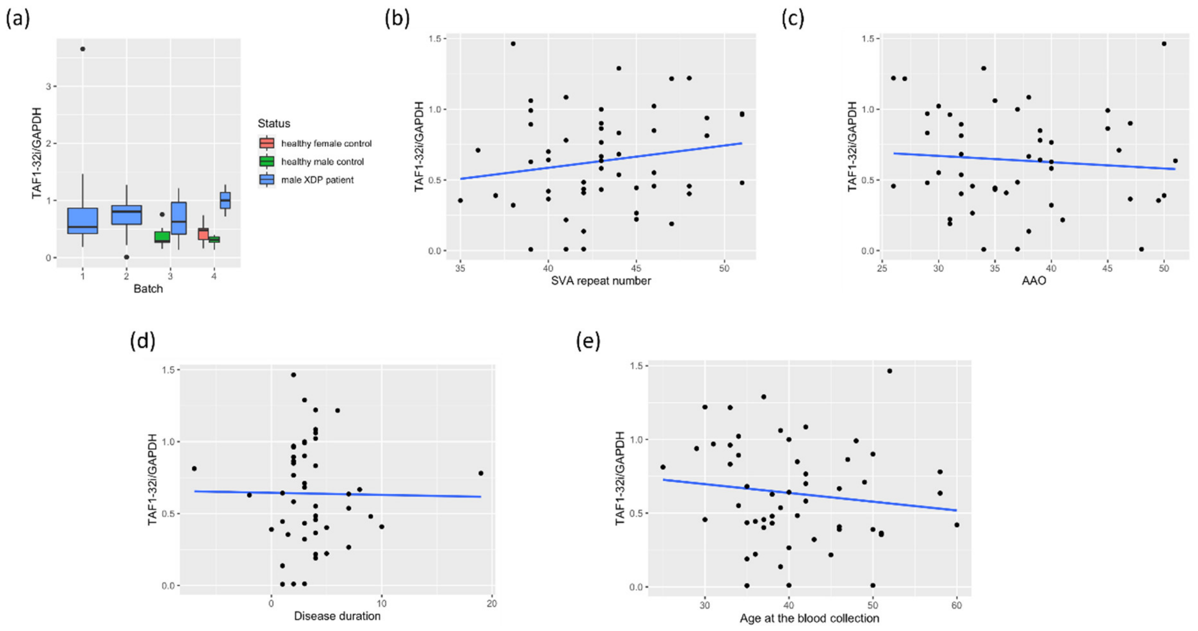

Figure S3. Correlation analysis between levels of *TAF1-32i* relative to *GAPDH* in blood-derived cDNA samples. (**a**) Corrected values of *TAF1-32i*/*GAPDH* for multiple independent batches; (**b**) linear correlation between relative *TAF1-32i* levels and the SVA repeat number in male patients with XDP; (**c**) linear correlation between relative *TAF1-32i* levels and age at disease onset (AAO); (**d**) linear correlation between relative *TAF1-32i* levels and disease duration; (**e**) linear correlation between relative *TAF1-32i* levels and age at blood collection.

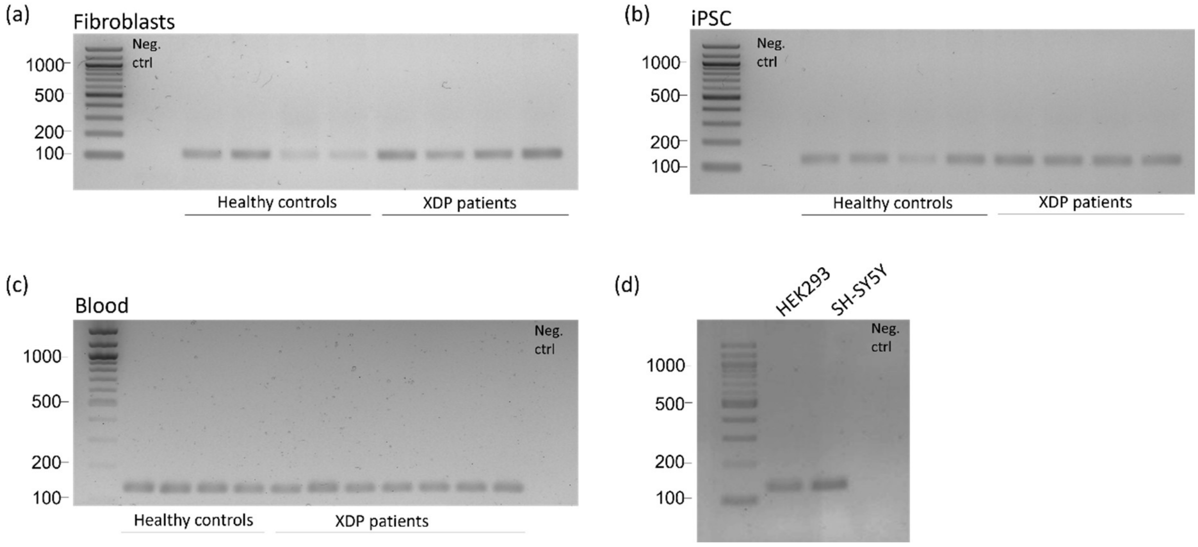

Figure S4. Agarose gels for *TAF1-32i* in various cell lines and tissues, using the same primers that were optimized for the qPCR reaction. (**a**) fibroblasts; (**b**) iPSC; (**c**) blood; (**d**) HEK293 and SH-SY5Y.
